# Increased insect herbivore performance under elevated CO_2_ is associated with lower plant defence signalling and minimal declines in nutritional quality

**DOI:** 10.1038/s41598-020-70823-3

**Published:** 2020-09-03

**Authors:** Scott N. Johnson, Jamie M. Waterman, Casey R. Hall

**Affiliations:** Hawkesbury Institute for the Environment, Western Sydney University, Locked Bag 1797, Penrith, NSW 2751 Australia

**Keywords:** Agroecology, Climate-change ecology, Plant ecology, Plant stress responses

## Abstract

Changes in insect herbivore performance under elevated atmosphere carbon dioxide concentrations e[CO_2_] are often driven by changes in the nutritional and defensive chemistry of their host plants. Studies addressing how the prolific pest cotton bollworm (*Helicoverpa armigera*) responds to e[CO_2_] show that performance usually declines, often associated with lower nutritional (e.g. nitrogen (N) concentrations) quality of host plants under e[CO_2_]. We investigated the impacts of e[CO_2_] on nutritional quality and anti-herbivore (jasmonate) defensive signalling in lucerne (*Medicago sativa*) when challenged by *H. armigera*. While foliar N decreased under e[CO_2_], other aspects of nutritional quality (soluble protein, amino acids, foliar C:N) were largely unaffected, potentially due to increased root nodulation under e[CO_2_]. In contrast, e[CO_2_] greatly reduced jasmonate signalling in *M. sativa* following *H. armigera* attack; jasmonic acid concentrations were *ca*. 56% lower in attacked plants grown under e[CO_2_]. Concurrent with this, relative growth rates of *H. armigera* were *ca.* 66% higher when feeding on e[CO_2_]-grown plants. In contrast with previous reports, which we meta-analytically summarise, we provide the first evidence that *H. armigera* performance can increase under e[CO_2_]. This may occur in plants, such as *M. sativa*, where e[CO_2_] has limited impacts on nutritional quality yet reduces jasmonate defence signalling.

## Introduction

With global populations expected to reach 11.2 billion by 2,100 there is an urgent need to ensure future food security, a challenge which is complicated by global climate change^1^. Invertebrate pests destroy enough food to feed a billion people a year^2^ which has, in part, fuelled interest in understanding which pests may become more problematic under predicted changes in the Earth’s climate^3^. In particular, unprecedented increases in atmospheric carbon dioxide (CO_2_) have the capacity to change plant chemistry which affect the susceptibility of crops to insect herbivores^4–6^.

The effects of elevated atmospheric CO_2_ concentration (e[CO_2_]) on plant nutritional and defensive chemistry, and their consequent effects on invertebrate herbivores, have received extensive attention^7–9^. In terms of nutritional quality, nitrogen availability is considered to be the limiting factor in insect herbivore diets^10^. Broadly speaking, e[CO_2_] causes foliar nitrogen (N) concentrations to decrease due to one or more processes, including dilution effects (i.e. relative to increased carbohydrate concentrations), reduced N uptake by roots, increased NH_3_ volatilization and reduced investment in the N-rich enzyme RUBISCO^11–14^. The impacts of e[CO_2_] on plant defences are less easily predicted, but some trends are emerging^5^. Plant defences against herbivorous arthropods are regulated by several phytohormonal pathways, including the jasmonic acid (JA), salicylic acid (SA) and ethylene signalling pathways^15,16^. Of these, the JA pathway is regarded as the master regulator of plant resistance to arthropod herbivores, especially chewing herbivores^17^. A growing number of studies, reviewed by Zavala, et al.^5^ and Ode, et al.^6^, suggest that e[CO_2_] downregulates constitutive and herbivore-induced activity of the JA signalling pathway.

Some pests, such as the cotton bollworm (*Helicoverpa armigera* Hübner), have now reached critical pest status because of their economic damage and rapid ability to invade new regions ^18^. In particular, *H. armigera* is a major global pest of diverse agricultural and horticultural crops causing upwards of US $ 7 billion annually in crop losses^19^. This has, in part, prompted research into which crops may be at risk from *H. armigera* under predicted atmospheric CO_2_ concentrations. To our knowledge, there are 10 published studies reporting the plant-mediated effects of e[CO_2_] on *H. armigera* across four plant families: Fabaceae^20,21^, Malvaceae^22–25^, Poaceae^26–28^ and Solanaceae^29^. Most of these studies reported that e[CO_2_] reduced many, but not all, *H. armigera* performance parameters^20–27^. Two studies, in contrast, reported that relative growth rates were unaffected by e[CO_2_]^28,29^. Where declines in performance have been reported this has largely been attributed to lower plant nutritional (i.e. nitrogen) quality under e[CO_2_]^20,21,23–25,27^.

Similar responses may not hold true for all plant types, however. In their meta-analysis, Robinson, et al.^4^ showed that e[CO_2_] decreased N by just 10% in legumes, which was substantially less than reductions in N concentrations in non-leguminous plants (– 17%). This most likely stems from the fact that legumes form associations with N-fixing bacteria in their root nodules and e[CO_2_] often increases nitrogen fixation^30,31^. Any reductions in N concentrations of legumes arising under e[CO_2_] may therefore be less extensive than in non-leguminous plants^4^. e[CO_2_] may even lead to increases in some foliar amino acid concentrations where it substantially stimulates N-fixation^32,33^. *Helicoverpa armigera* attacks several legumes, including lucerne (alfalfa) (*Medicago sativa* L.), but to our knowledge no studies have yet addressed how e[CO_2_] affects *H. armigera* when feeding on this important forage legume.

The main objective of this study was to experimentally determine how e[CO_2_] affected *M. sativa* traits (biomass, root nodule abundance and density), nutritional chemistry (foliar carbon, nitrogen, protein and amino acid concentrations), defensive (JA) signalling and establish whether such changes were associated with changes in the growth rates of *H. armigera*. Our main hypothesis is that e[CO_2_] has negligible impacts on *M. sativa* nutritional status but depresses JA activity, which results in enhanced performance of *H. armigera*. Additionally, we conducted a simple a meta-analysis of previous studies to quantify overall effect of e[CO_2_] on the numerous *H. armigera* performance parameters reported to date.

## Results

**Figure 1:**
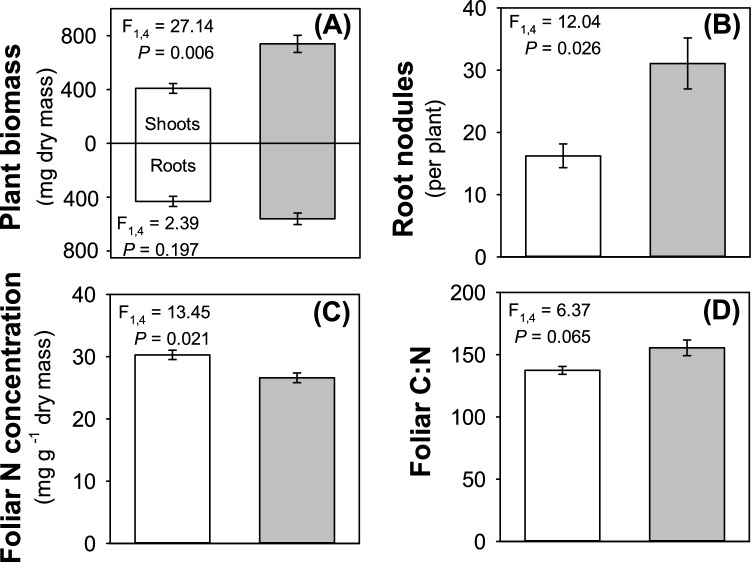
Impacts of a[CO_2_] (white bars) and e[CO_2_] (grey bars) on **(A)** plant biomass, **(B)** root nodule abundance, **(C)** foliar nitrogen concentration and **(D)** foliar carbon concentration. Mean ± standard error shown (N = 24 for **(A)** and **(B)**; N = 12 for **(C)** and **(D)**).

As detailed in the Methods, ambient CO_2_ (a[CO_2_]) and e[CO_2_] conditions were maintained at 400 and 640 ppm, respectively, and the experiment ran for eight weeks. Total plant biomass increased by an average of *ca*. 55% under e[CO_2_] (F_1,4_ = 11.30, *P* = 0.028), with this increase being reflected in the shoots (Fig. 1A). Root mass was unaffected by e[CO_2_] but root nodule abundance almost doubled under e[CO_2_] (Fig. 1B). In accordance with there being no change in root mass, but an increase in nodule numbers, there was an increase in nodule density on the roots of plants grown under e[CO_2_] compared to a[CO_2_] (58.9 and 41.0 nodules g^-1^ dry root mass, respectively) (F_1,4_ = 14.72, *P* = 0.019).

**Figure 2:**
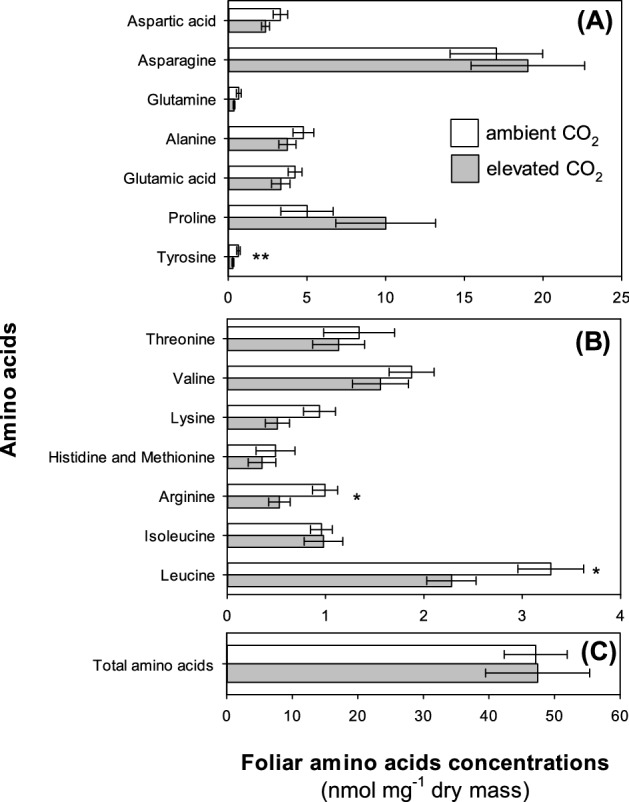
Impacts of a[CO_2_] (white bars) and e[CO_2_] (grey bars) on **(A)** total amino acids, **(B)** non-essential and **(C)** essential amino acids in the foliage. Mean ± standard error shown (N = 14).

There was a significant decline (– 12%) in foliar concentrations of total nitrogen (Fig. 1C) under e[CO_2_], but no changes in carbon concentrations (F_1,4_ = 0.01, *P* = 0.929) resulting in a small but non-significant increase in foliar C:N ratio (Fig. 1D). Despite the decrease in nitrogen concentrations overall, soluble protein concentrations were very similar in plants grown under a[CO_2_] and e[CO_2_] conditions: 68.15 ± 2.65 and 66.45 ± 3.58 mg g^−1^, respectively (mean ± standard error, N = 12; F_1,4_ = 0.04, *P* = 0.846). Concentrations of total amino acids were also unaffected by CO_2_, (Fig. 2A; F_1,4_ = 0.01, *P* = 0.973). Both non-essential (Fig. 2B) and essential (Fig. 2C) amino acids were similarly unaffected by CO_2_ (F_1,4_ = 0.28, *P* = 0.624 and F_1,4_ = 3.41, *P* = 0.139, respectively), although there was a small decline in concentrations of tyrosine, arginine, leucine and phenylalanine under e[CO_2_] (Fig. 2C and Table S2).

**Figure 3:**
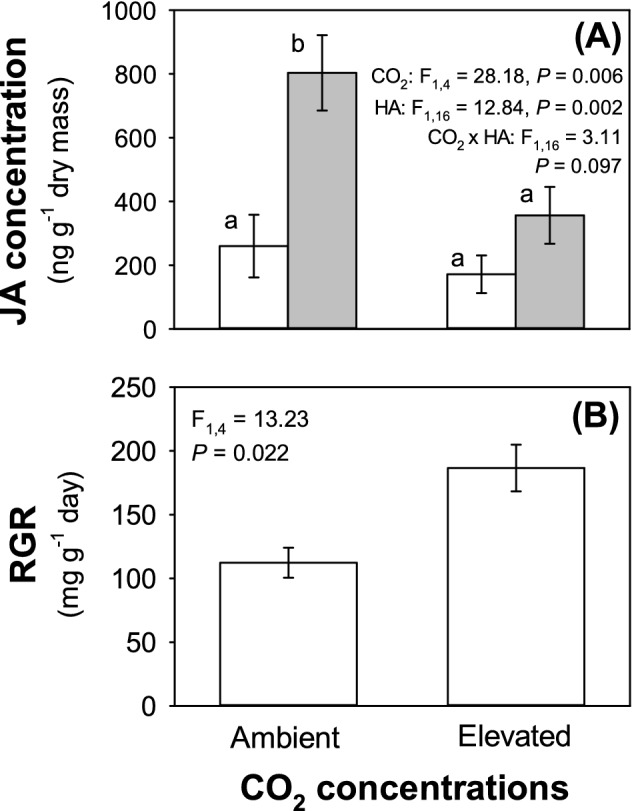
Impacts of a[CO_2_] and e[CO_2_] on **(A)** JA concentrations when plants are challenged (grey bars) and unchallenged (white bars) by *H. armigera* and **(B)** RGR of *H. armigera*. Mean ± standard error shown (N = 6 for **(A)**; N = 12 for **(B)**).

Under a[CO_2_], herbivory by *H. armigera* triggered a sharp increase (ca. + 209%) in JA concentrations; this was not seen under e[CO_2_] and JA concentrations remained at similar levels as unchallenged plants grown under either CO_2_ regime (Fig. 3A). Concurrent with this, *H. armigera* developed significantly more rapidly (ca. 66% gain in mass per day) under e[CO_2_] compared to those feeding on plants grown at a[CO_2_] (Fig. 3B).

**Figure 4:**
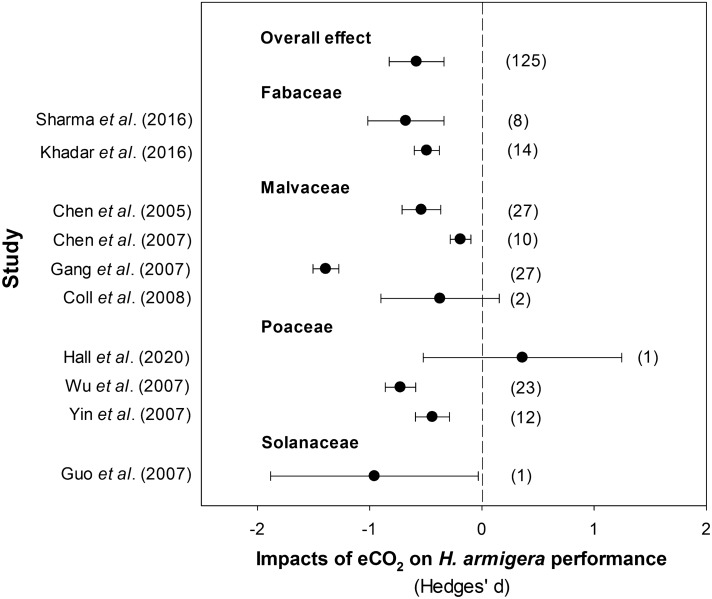
Meta-analytical summary of previous studies addressing the plant-mediated impacts of e[CO_2_] on *H. armigera* performance. Effect size (95% confidence interval) shown with the number of observations reported in parentheses. Points to the left indicate significant decreases in performance under e[CO_2_] relative to *H. armigera* feeding under a[CO_2_]. Studies: Sharma et al. (2016)^20^, Khadar et al. (2014)^21^, Chen et al. (2005)^23^, (2007)^22^, Gang et al. (2007)^25^, Coll et al. (2008)^24^, Hall et al. (2020)^28^, Wu et al. (2006)^27^, Yin et al. (2010)^26^ and Guo et al. (2012)^29^.

The meta-analytical comparison of the ten studies investigating the effects of e[CO_2_] on *H. armigera* performance (125 observations) quantitatively confirmed that there was an overall negative impact of e[CO_2_] on *H. armigera* performance and that no studies found positive effects (Fig. 4). This took into account all of the 125 performance parameters that were measured, including those that were unaffected or promoted by e[CO_2_]. The studies used a range (550–750 ppm) of CO_2_ concentrations for e[CO_2_] treatments with the average being 699.50 + 22.45 ppm (mean ± standard error). The e[CO_2_] level used in the current study was comparable with this (8.5% lower).

## Discussion

Our results show that e[CO_2_] accelerates the growth rates of *H. armigera* when feeding on *M. sativa*, to our knowledge the first example of *H. armigera* benefitting from e[CO_2_]. The meta-analytical summary confirmed quantitatively that, taken together, existing studies indicate that e[CO_2_] causes declines in *H. armigera* performance. We suggest that the increase in *H. armigera* growth rates in e[CO_2_] we observed was associated with the suppressed JA response, which potentially triggers downstream plant defences, and the relatively modest impacts of e[CO_2_] on host plant nutritional quality compared to previous studies.

While we did observe a significant decline in total N concentrations in e[CO_2_], we saw no changes in soluble protein or total amino acids and only marginal (non-significant) increases in foliar C:N. The decline in total N concentrations might, in part, be due to the lower concentrations of the essential (arginine, leucine and phenylalanine) and non-essential (tyrosine) under e[CO_2_]. This might not necessarily represent a decline in nutritional quality however, and may even reflect lower levels of plant defences such as flavonoids, which are key defences in *M. sativa*. Flavonoids are a part of the phenylpropanoid pathway and are ultimately derived from phenylalanine and tyrosine^34^. Moreover, arginine plays a critical role in nitric oxide metabolism, which regulates defence responses to biotic stress^35^. In addition, given that protein concentrations were unaffected by CO_2_, this suggests that decreases in these specific amino acids was not substantial enough to hinder protein synthesis. Collectively, this suggests that e[CO_2_] impacts on nutritional quality was limited.

The greater abundance of root nodules housing N-fixing bacteria under e[CO_2_] may have contributed to N levels and therefore limited the negative impacts of e[CO_2_] on nutritional quality. e[CO_2_] can increase N-fixation via several mechanisms, including larger numbers of N-fixing bacteria^36^, increased nodulation^37^ and enhanced nitrogenase activity^38^. This effect depends, however, on other factors such as nutrient availability^39^ and air temperature^31^. The two studies that used legumes to investigate the impacts of e[CO_2_] on *H. armigera* reported declines in total N (–18 to 25%)^21^ or protein content (–18 to 28%)^20^ in the foliage. In contrast, we observed more modest declines in total N (–12%) and almost no change in protein (–2.5%) or amino acid (+ 0.6%) concentrations. The effects of e[CO_2_] on root nodulation and amino acid concentrations were not reported in the other studies, but it would seem unlikely that N-fixation was being stimulated in these experiments. It seems reasonable to assume in the current study that the nutritional quality of the *M. sativa* did not appreciably decline under e[CO_2_] in the current study using the Lepidopteran *H. armigera*. It remains possible, however, that herbivores with different feeding guilds (e.g. aphids) and other *M. sativa* genotypes might have been affected differently.

While we did not quantify specific defensive metabolites, our results are consistent with the findings of Guo, et al.^29^ who also reported that e[CO_2_] depressed JA responses against *H. armigera* in tomato. Interestingly, this did not affect the relative growth rates of *H. armigera.* Guo, et al.^29^ speculated that this may have been due to lower N concentrations of plants grown under e[CO_2_], which we suggest did not occur to the same extent in the current study. The combination of depressed JA without substantive changes in nutritional quality under e[CO_2_] therefore most likely explains why *H. armigera* performed better on lucerne grown under e[CO_2_]. There is a small possibility that increased RGR was due to increased leaf temperature under e[CO_2_], though presumably this would have also occurred in studies reporting reduced performance under e[CO_2_] so this seems unlikely.

The exact mechanism by which e[CO_2_] affects JA in other systems remains unclear^5^. One possibility is that JA patterns operate via circadian regulation, peaking during the day when photosynthesis rates are highest and intercellular CO_2_ is lowest^40^. Intracellular CO_2_ is lowest during the night which is linked to lower JA concentrations, so higher CO_2_ may more generally result in lower JA concentrations^5^. Moreover, e[CO_2_] has been linked to enhanced SA concentrations, which often has an antagonistic relationship with the JA pathway^41^.

In reporting these findings, we aim to highlight that the effects of e[CO_2_] on this important insect herbivore cannot be universally assumed to be negative or neutral even though this has consistently been the case in previous studies. In particular, compromised defence signalling (potentially leading to lower downstream production of defences) in e[CO_2_] relative to a[CO_2_], together with relatively modest declines in nutritional quality may explain enhanced *H. armigera* performance in some instances.

## Methods

### Chambers and environmental conditions

The study was conducted in six naturally lit glasshouse chambers (3 × 5 × 3 m; width × length × height) with UV transparent plexiglass (6 mm thick) walls and roof. Air temperature was regulated at 25 °C (± 0.25 °C). Humidity was controlled at 60% (± 6%). Three of the chambers were maintained at ambient CO_2_ (aCO_2_; 400 µmol mol^−1^) and three chambers at eCO_2_ (640 µmol mol^−1^). The elevated concentration reflects low-mid predictions for 2,100 depending on scenario^42^. Environmental conditions were monitored continuously throughout the experiment and temperature readings were verified with portable temperature loggers.

### Experimental procedure

Lucerne (*M. sativa* cv. Sequel) was grown from seed (Seedmark, Adelaide, Australia) in 72 pots (70 mm diameter) filled with 700 g of native soil mix (Turtle Nursery and Landscape Supplies; see Table S1 for compositional details). These were distributed evenly between the six chambers, such that 36 plants were grown under either a[CO_2_] or e[CO_2_] (12 plants per chamber). Plants were watered with *ca*. 70 mL of tap water three times a week. After seven weeks, herbivores were applied to 12 plants grown under a[CO_2_] or e[CO_2_] (i.e. four plants in each of the six chambers). Specifically, *H. armigera* eggs (supplied by CSIRO, Narrabri) were individually hatched and reared on an artificial diet ^43^ at 20ºC under a 15:9 h photoperiod (L:D) for 7 days. One larva was weighed and applied to the base of the plants. White mesh (organza) bags (125 × 170 mm) were applied tightly around the rim of all pots (including herbivore free plants) to retain herbivores on their allocated plants. Seven days later, the herbivores were removed and reweighed before removing plants from the soil with root washing in water. Relative growth rates (RGR) were calculated [(change in mass / initial mass) / days]. The number of active (pink) root nodules was recorded for all of the herbivore-free plants. Roots and shoots from these plants were then snap frozen, freeze dried, ground and weighed prior to chemical analysis. All of the chemical analysis was conducted on ground tissue from herbivore-free plants using a sub-sample of the collective foliar material from each plant.

### Chemical analysis

Twelve plant samples, selected at random across chambers, for both CO_2_ treatments were used to determine total carbon, nitrogen and soluble protein concentrations in ground foliage. Carbon and nitrogen were quantified (using *ca.* 6 mg material) with an elemental combustion analyser (FLASH EA 112 Series CHN analyser, Thermo-Finnigan, Waltham, MA, USA). For soluble protein analysis, modified from Jones, et al.^44^, 1 mL of 0.1 M NaOH was added to *ca.* 23 mg of material and homogenised at 25 °C for 30 min. The mixture was then centrifuged at 12,000 rpm  for 5 min. The supernatant was removed and added to a clean microtube. A 1:4 dilution of each extract was made and dilutions were measured in technical triplicate on a on CLARIOstar High Performance Monochromator multimode microplate reader (BMG labtech, Offenburg, Germany) using the Bradford assay modified for a 96-well plate^44,45^. Protein concentrations were calculated using a standard curve of bovine serum albumin.

Fourteen plant samples, selected at random across chambers, for both CO_2_ treatments were used for amino acid analysis. Soluble amino acids were extracted from *ca.* 75 mg of foliar tissue with 525 μl 80% methanol, simultaneously heated and vortexed at 50 °C/850 rpm respectively. Samples were centrifuged and the supernatant filtered through 0.22 μm pore size nylon membrane. Underivatised amino acids were separated by reverse-phase high-performance liquid chromatography (HPLC) using an Agilent 1260 Infinity HPLC system equipped with an Agilent Poroshell 120 EC-C18 column (4.6 × 150 mm, 2.7 µm). Using a flow rate of 0.6 mL/min and an injection volume of 7 µl, analyte peaks were detected with a Corona charged aerosol detector (CAD; Corona CAD veo; Thermo Fisher Scientific Inc.) and eluted using two mobile phases (Solvent A: 0.4% heptafluorobutyric acid and 0.02% trifluoroacetic acid (TFA) in distilled water, Solvent B: 0.1% TFA in acetonitrile, modified from Furota, et al.^46^. Amino acid standards (0, 0.125 and 2 µmol 1^–1^) containing 16 amino acids were used to calibrate the analysis; arginine, histidine, isoleucine, leucine, lysine, methionine, phenylalanine, threonine and valine are essential (i.e. unable to be synthesized by insects de novo) and seven are non-essential; alanine, asparagine, aspartic acid, glutamic acid, glutamine, proline and tyrosine.

The procedures used for phytohormone analysis have previously been described in Hall et al. (2020)^28^. Jasmonic acid (JA) was analysed in six samples, selected at random, for combinations of CO_2_ and herbivore treatments (24 in total). Samples were extracted using the Bligh-Dyer method^47^ to remove interfering compounds. Ground leaf material (ca. 50 mg) was mixed with 500 µL of 70% methanol and 100 ppb of deuterated JA (d5-JA) as internal standard. Samples were then mixed for 30 min at 4 ºC in a rotator mixer, after which 180 µl of chloroform was added and samples vortexed for 30 s. The last step was repeated and then 200 µl of water was added, samples were then centrifuged for 10 min at 6,000 rpm at room temperature. The water/methanol solution was pipetted to a clean 2 ml Eppendorf tube and filtered using a 0.22 µm PTFE syringe filter. The extracts were analysed using an Acuity Ultra Performance Liquid Chromatography (UPLC) coupled to a Xevo triple quadrupole mass spectrometer (Waters Corporation, Milford, USA). Five microliters from each sample were injected into a 2.1 mm × 50 mm × 1.7 µm, C18 reverse phase column. The mobile phase consisting of water (A) and acetonitrile (B) both containing 0.1% (v/v) formic acid was passed through the column at a constant flow rate of 0.6 mL min^−1^ over a linear gradient (A%, t min): 80% A at 0 min; 50% A at 2 min; 0% A and 2.1 min. JA was detected by Electrospray ionization tandem mass spectrometry (ESI–MS/MS) in negative ion mode. Identification of JA was determined based on the fragmentation pattern of an authentic JA standard. JA was quantified using a calibration curve of the JA standard which was adjusted for sample recovery based on the concentration of the internal standard. Final JA concentrations were standardised by dry weight of the sample. The internal standard, d5-JA, was purchased from CDN Isotopes (Quebec, Canada). HPLC grade methanol, chloroform, and JA were purchased from Sigma-Aldrich (MO, USA).

### Statistical analysis

One-way ANOVAs with CO_2_ regime as the fixed factor were used to analyse most plant responses (biomass, nodule number and density, C, N, protein and amino acid concentrations) and herbivore RGR. For foliar JA concentrations a two-way ANOVA (CO_2_ × herbivory) was used with Fisher’s LSD test applied to determine differences between specific treatments. To avoid pseudoreplication, chamber was included as a block term with three chambers replicating each CO_2_ regime. Log transformations were applied for nodule number and nodule density to meet assumptions of normality and heteroscedasticity. Logit transformations were applied to C concentrations, foliar C:N ratio, some amino acids (see Table S2) for the same reasons. Satisfactory transformation was not possible in one instance and a Kruskal–Wallis test was applied (see Table S2 for details). Analysis was conducted using Genstat (version 18, VSN International, Hemel Hempstead, UK).

### Meta-analysis

Original research papers were identified via searches on Web of Science and BIOSIS on 8 April 2020 using the search terms ‘carbon dioxide’ AND ‘*Helicoverpa armigera*’ together with ‘CO_2_’ AND ‘*Helicoverpa armigera*’. After removal of duplicates, 54 records were identified for initial screening (Fig. S1). Examination of titles and/or abstracts resulted in 36 studies being excluded on the grounds that they were not relevant or did not contain data (e.g. reviews). The remaining 18 full-text articles were assessed for eligibility, resulting in the removal of eight studies for specific non-compliance issues (listed in Fig. S1). Performance were parameters classified as abundance, feeding efficiency, growth/development, mortality/survival and reproduction were used. Numerical data were extracted from graphical figures using DigitizeIt (v2.3.3; Bormisoft, Braunschweig, Germany). For performance parameters where higher numerical values indicated poorer herbivore performance (e.g. mortality), a negative sign was applied to the value. We used responses measured on plants that excluded other treatments (e.g. transgenic Bt or silicon supplementation) and selected one performance parameter in the few cases where responses were essentially duplicates (e.g. relative growth rate and mean relative growth rate).

Meta-analysis were conducted using the package *metafor*^48^ in the R statistical platform. The effect size (Hedges’ d) was calculated for each pair of performance responses (i.e. at a[CO_2_] and e[CO_2_]). Where more than one e[CO_2_] level was applied, both levels were included as separate entries. This measure of effect size compares two means using a pooled standard deviation and bias correction and reflects the number of standard deviations by which the means differ^49^. Positive values arise when herbivores performed better on plants grown in e[CO_2_] compared to control plants (a[CO_2_] plants) whereas negative values indicate the opposite (i.e. they perform worse on e[CO_2_] grown plants).

## Supplementary information

Supplementary Information.

## Data Availability

All meta-analysis and empirical data are posted on the figshare repository https://doi.org/10.6084/m9.figshare.12480068.
