# Author Correction: Increased insect herbivore performance under elevated CO_2_ is associated with lower plant defence signalling and minimal declines in nutritional quality

**DOI:** 10.1038/s41598-020-78543-4

**Published:** 2020-12-07

**Authors:** Scott N. Johnson, Jamie M. Waterman, Casey R. Hall

**Affiliations:** Hawkesbury Institute for the Environment, Western Sydney University, Locked Bag 1797, Penrith, NSW 2751 Australia

Correction to: *Scientific Reports* https://doi.org/10.1038/s41598-020-70823-3, published online 03 September 2020

This Article contains errors in Figure 2, where the panels (A), (B) and (C) are in an incorrect order, and one of the results given in the Article, the effect of elevated CO_2_ on phenylalanine concentrations, is omitted in the Figure.

The correct version of Figure 2 appears below as Figure 1.

**Figure 1:**
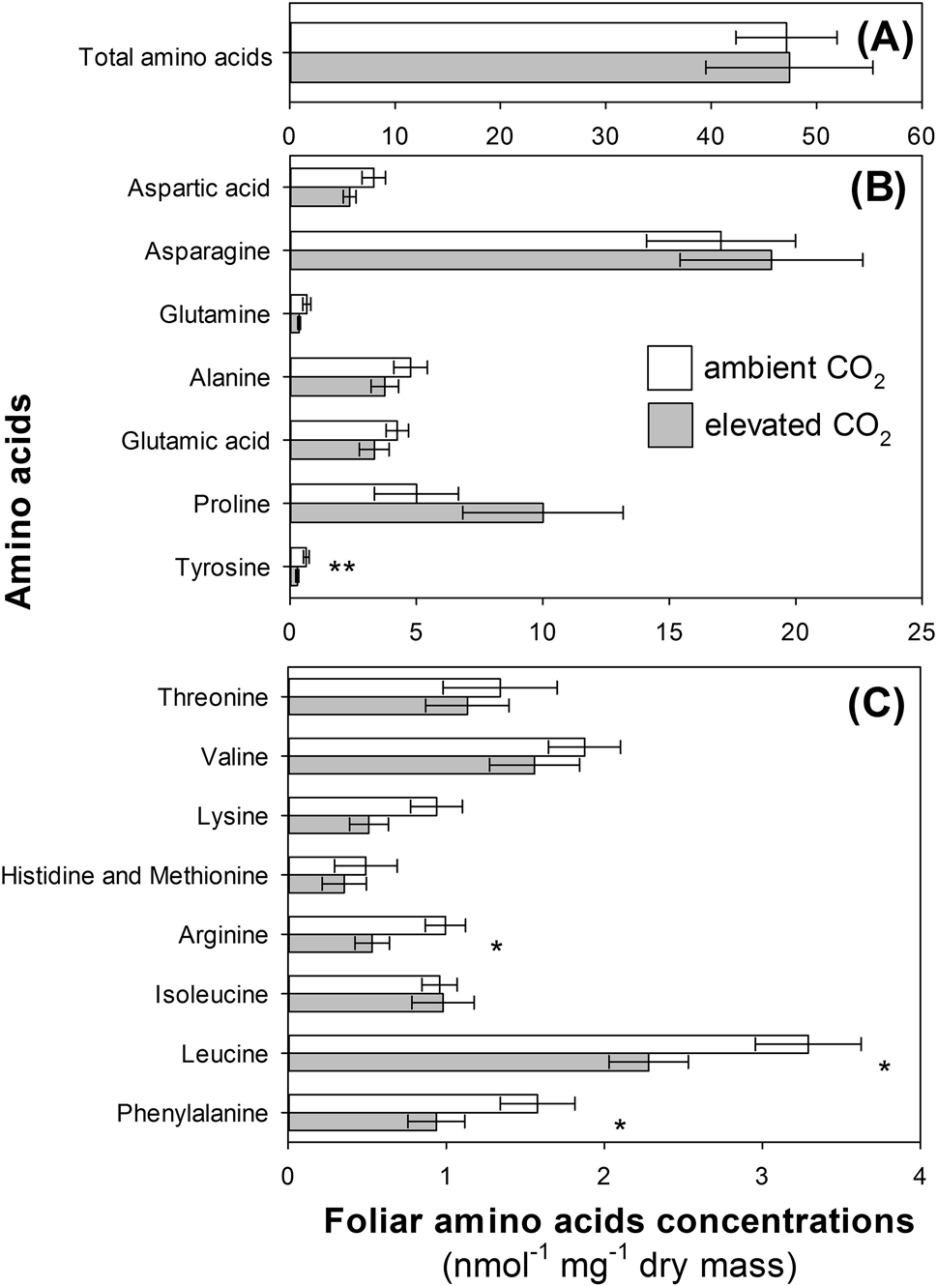
A correct version of the original Figure 2.

