# Supplementary material for: Increased insect herbivore performance under elevated CO2 is associated with lower plant defence signalling and minimal declines in nutritional quality
Source: Sci Rep. 2020 Sep 3;10:14553. doi: 10.1038/s41598-020-70823-3 (PMC7471906; doi:10.1038/s41598-020-70823-3)
Supplement: Supplementary file 1 — Supplementary Information. [file 41598_2020_70823_MOESM1_ESM.docx]

**Supplemental Material**

**Increased insect herbivore performance under elevated CO_2_ is associated with lower plant defence signalling and minimal declines in nutritional quality**

Scott N. Johnson, Jamie M. Waterman and Casey R. Hall

*Hawkesbury Institute for the Environment, Western Sydney University, Locked Bag 1797, Penrith, NSW 2751, Australia*

*Corresponding author: [Scott.Johnson@westernsydney.edu.au](mailto:Scott.Johnson@westernsydney.edu.au)

**Fig. S1.** PRISMA (Preferred Reporting Items for Systematic reviews and Meta Analyses) flow diagram reporting identification, screening and eligibility determination for studies included in the meta-analysis.

Studies included in quantitative synthesis (meta-analysis)
(n = 10)

Studies included in qualitative synthesis
(n = 10)

Full-text articles assessed for eligibility
(n = 18)

Records excluded
(n = 36)

Records screened
(n = 54)

Records after duplicates removed
(n = 54)

Additional records identified through other sources
(n = 0)

## Identification

## Eligibility

## Included

## Screening

Records identified through database searching
(n = 79)

Full-text articles excluded, with reasons
(n = 8)

Conducted using artificial diet (i.e. direct effects of CO_2_ gas) = 2

No CO_2_ involved = 1

Herbivore performance not measured = 4

Review article = 1

**Table S1**. Chemical composition of the soil used in the study (N = 2). Analysis conducted on oven dried (40ºC) soil sieved < 2mm by Environmental Analysis Laboratory, Southern Cross University, Australia. Specific methods described in Rayment and Lyons (2011).

| **Method** | **Nutrient / Property** | **Units** | **Mean** | **Standard Error** |
| --- | --- | --- | --- | --- |
| Morgan 1 | Phosphorus | mg/kg | 93.00 | 16.00 |
| KCl | Nitrate Nitrogen | mg/kg | 0.71 | 0.01 |
|  | Ammonium Nitrogen | mg/kg | 3.43 | 0.20 |
| Base Saturation Calculations | Calcium | % | 73.71 | 0.65 |
|  | Magnesium | % | 12.07 | 0.09 |
|  | Potassium | % | 10.38 | 0.43 |
|  | Sodium - ESP | % | 3.74 | 0.14 |
| CaCl_2_ | Silicon (bioavailable) | units | 69.40 | 6.40 |
|  | Soil pH (CaCl_2_) | mg/kg | 6.04 | 0.12 |
| LECO IR Analyser | Total Carbon | % | 4.32 | 0.22 |
|  | Total Nitrogen | % | 0.09 | 0.01 |

Rayment GE, Lyons DJ (2011) Soil Chemical Methods - Australasia. Australian soil and land survey handbooks ; 3., vol Accessed from <https://nla.gov.au/nla.cat-vn4854518>. CSIRO Publishing, Collingwood, VIC

**Table S2**. One-way ANOVA tests and a Kruskal-Wallis test^2^ conducted on individual foliar amino acids. Significant impacts of e[CO_2_] on amino acids highlighted in bold.

| **Amino acid type** | **Amino acid** | **Statistical Results** | |
| --- | --- | --- | --- |
|  |  | F_1,4_ | *P* |
| Non-essential | Alanine | 0.57 | 0.493 |
|  | Asparagine^1^ | 0.07 | 0.807 |
|  | Aspartic acid | 1.45 | 0.295 |
|  | Glutamic acid | 2.11 | 0.220 |
|  | Proline | 4.75 | 0.095 |
|  | **Tyrosine** | **21.92** | **0.009** |
| Essential | **Arginine** | **13.45** | **0.021** |
|  | Histidine and methionine^2^ | H = 15.89 | 0.368 |
|  | Isoleucine | 0.02 | 0.892 |
|  | **Leucine** | **14.54** | **0.019** |
|  | Lysine | 4.36 | 0.105 |
|  | **Phenylalanine** | **8.05** | **0.047** |
|  | Threonine^1^ | 0.27 | 0.632 |
|  | Valine | 1.79 | 0.252 |

^1^ Logit transformations applied prior to analysis ^2^Kruskal-Wallis Test used as transformation was not possible.
